# Natural Flavonoids as Potential Angiotensin-Converting Enzyme 2 Inhibitors for Anti-SARS-CoV-2

**DOI:** 10.3390/molecules25173980

**Published:** 2020-09-01

**Authors:** Muchtaridi Muchtaridi, M. Fauzi, Nur Kusaira Khairul Ikram, Amirah Mohd Gazzali, Habibah A. Wahab

**Affiliations:** 1 Department of Pharmaceutical Analysis and Medicinal Chemistry, Faculty of Pharmacy, Universitas Padjadjaran, Jl Raya 21.5, Bandung-Sumedang 45363, Indonesia; muhammad18254@mail.unpad.ac.id; 2 Institute of Biological Sciences, Faculty of Science, Universiti Malaya, Kuala Lumpur 50603, Malaysia; nkusaira@um.edu.my; 3 Centre for Research in Biotechnology for Agriculture (CEBAR), Universiti Malaya, Kuala Lumpur 50603, Malaysia; 4 Department of Pharmaceutical Technology, School of Pharmaceutical Sciences, Universiti Sains Malaysia, Gelugor 11800, Penang, Malaysia; amirahmg@usm.my; 5 Pharmaceutical Design and Simulation Laboratory, School of Pharmaceutical Sciences, Universiti Sains Malaysia, Gelugor 11800, Penang, Malaysia

**Keywords:** ACE2, COVID-19, flavonoid, coronavirus

## Abstract

Over the years, coronaviruses (CoV) have posed a severe public health threat, causing an increase in mortality and morbidity rates throughout the world. The recent outbreak of a novel coronavirus, named severe acute respiratory syndrome coronavirus 2 (SARS-CoV-2) caused the current Coronavirus Disease 2019 (COVID-19) pandemic that affected more than 215 countries with over 23 million cases and 800,000 deaths as of today. The situation is critical, especially with the absence of specific medicines or vaccines; hence, efforts toward the development of anti-COVID-19 medicines are being intensively undertaken. One of the potential therapeutic targets of anti-COVID-19 drugs is the angiotensin-converting enzyme 2 (ACE2). ACE2 was identified as a key functional receptor for CoV associated with COVID-19. ACE2, which is located on the surface of the host cells, binds effectively to the spike protein of CoV, thus enabling the virus to infect the epithelial cells of the host. Previous studies showed that certain flavonoids exhibit angiotensin-converting enzyme inhibition activity, which plays a crucial role in the regulation of arterial blood pressure. Thus, it is being postulated that these flavonoids might also interact with ACE2. This postulation might be of interest because these compounds also show antiviral activity in vitro. This article summarizes the natural flavonoids with potential efficacy against COVID-19 through ACE2 receptor inhibition.

## 1. Introduction

**Figure 1:**
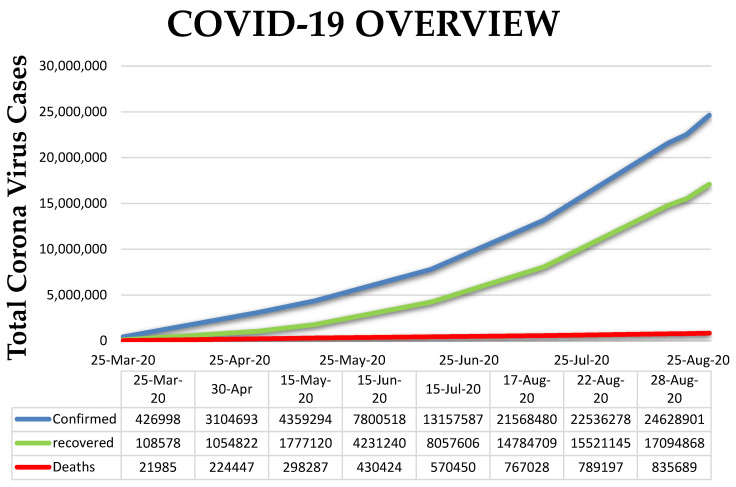
The rise in active cases of coronavirus [2].

Severe acute respiratory syndrome coronavirus 2 (SARS-CoV-2), which is the causative agent of Coronavirus Disease 2019 or COVID-19, triggered a pandemic affecting over 215 countries and territories around the world [1,2]. As of August 2020, there are more than 23 million cases worldwide with over 800,000 deaths, indicating that the virus is highly infectious with its pathogenicity being a global health threat [3,4,5]. The number of positive cases and deaths due to COVID-19 continues to increase rapidly and, due to the unavailability of effective drugs, recovery is lagging (Figure 1) [2,6,7]. Thus, the search for new drugs to overcome this disease needs to be urgently intensified [2,8].

SARS-CoV-2, which causes severe respiratory syndrome in humans, is a positive-strand RNA virus. The virus replication cycle begins with the entry of the virus into the human body by attaching to the host cellular receptor angiotensin-converting enzyme 2 (ACE2), assisted by a protein spike (S), followed by the release of the virus genome material into the host cell [9]. The viral genome contains two overlapping polyproteins (polyprotein 1a and polyprotein 1ab), which are cleaved by Mpro (the main protease) into 16 non-structural proteins, which are then translated into structural (STR proteins) and non-structural proteins (non-STRs). This is followed by virus assembly, which releases virions from the infected cells through exocytosis [10,11].

The angiotensin-converting enzyme (ACE)-related carboxypeptidase, ACE2, is a type I integral membrane protein of 805 amino acids containing one HEXXH-E zinc-binding consensus sequence [12]. ACE2 is involved in regulating cardiac function and is also a functional receptor for the coronavirus that causes acute respiratory syndrome (SARS). ACE2 receptors are the largest target of SARS-CoV-2 because they play an important role in the transmission of viruses to alveolar cells [13]. Inhibition or regulation of ACE2 receptors may potentially be effective in the treatment of COVID-19. COVID-19 is currently being treated with anti-infective drugs such as antimalarial drugs (chloroquine, hydroxychloroquine [14,15,16,17], antiviral drugs (remdesivir [18], saquinavir [19], favipiravir [20], lopinavir [21], ribavirin [22], and oseltamivir), and certain immunosuppressive drugs such as tocilizumab [23]. Tocilizumab was approved by the Food and Drug Administration (FDA) to manage cytokine release syndrome (CRS) in patients receiving chimeric antigen receptor T-cell therapy. This drug was shown to reduce toxicity and improve immune-related toxicity [24,25]. Tocilizumab can block the activity of proinflammatory interleukin-6 (IL-6), which is involved in the pathogenesis of pneumonia that causes death in COVID-19 patients [26]. However, to date, we are still waiting for the results of the ongoing phase 3 clinical trial that might support and prove the effectiveness of these drugs in treating patients with SARS-CoV-2 infection. For example, Wang et al. (2020) conducted a randomized study on the use of placebo-controlled and intravenous remdesivir in 10 hospitals in Hubei, China [27]. The study found that intravenous remdesivir did not significantly increase the time for clinical improvement, the mortality, or the time for virus clearance in patients with serious SARS-CoV-2 compared to placebo. However, hydroxychloroquine or chloroquine with or without azithromycin did not enhance clinical status at 15 days [28]. In an effort to find new therapies for COVID-19, natural product sources are also being explored and re-evaluated for their activity against this deadly virus [24]. 

Natural compounds with high bioavailability and low cytotoxicity are the most efficient candidates [29]. Flavonoids are structurally heterogeneous, polyphenolic compounds present in high concentrations. Flavonoids are natural products found in many plants, and they play an important role in plant physiology; they were intensively investigated for having bioactivity beneficial to health, such as anti-inflammatory [30], anticancer [31], antioxidant [32], anti-lipogenic [33], metal-chelating [34], antimicrobial [35], and antiviral [36] properties. More than 2000 plant-derived flavonoids have been identified. Bioactive compounds from flavonoid derivatives are valuable for the development of drugs and as additional therapies for these infections. Other flavonoids including flavones and flavonoids were investigated for having antiviral potential, and many of them showed significant antiviral responses in both in vitro and in vivo studies. Naringenin and hesperetin (flavanon), hesperidin (flavanonone glycoside), baicalin and neohesperidin (flavone glycoside), nobiletin (*O*-methylation), scutellarin (flavone), nicotinamin (nonproteinogenic amino acids), and glycyrinodin (methylated-eminin-1,3,8-trihydroxyanthraquinone)are amongst natural ACE2 inhibitors [37,38,39]. This review focuses on the prospect of utilizing flavonoids as potential treatment for SARS-CoV-2 infection. 

## 2. Methods

This review was based on the literature obtained from PubMed and Google Scholar using 15 keywords. The results of the initial search strategy were firstly filtered by title and abstract. The full text of the relevant articles was examined for inclusion and exclusion criteria. When an article reported duplicate information from the same source, the information of the two reports was combined to obtain the complete data but was only counted as one case. A list of selected references from papers taken was used to further identify relevant citations. For the purpose of this review, the research focused on seven key words, namely, “coronavirus”, “angiotensin-converting enzyme”, “angiotensin converting enzyme II of coronavirus”, “angiotensin-converting enzyme II inhibitor CoV”, “natural compounds ACE and ACEII inhibitors enzyme II of coronavirus”, “flavonoid as antiviral, antioxidant, antiinflammation”, and “flavonoid as ACE2 inhibitor

## 3. Severe Acute Respiratory Syndrome Coronavirus 2 (SARS-CoV-2)

SARS-CoV-2 initially appeared as part of a major outbreak of respiratory disease centered in Hubei Province, China. It was identified as a novel type of coronavirus. Coronaviruses belong to the large and enveloped *Coronaviridae* family under the *Nidovirales* order of viruses with positive-stranded crown-like RNA [40,41]. The viral genome is 27 to 32 kb in size and is the largest virus among all RNA viruses [6,42]. There are six types of coronaviruses, namely, alphacoronavirus 229E, alphacoronavirus NL63, betacoronavirus OC43, HKU1 betacoronavirus, severe acute respiratory illness coronavirus (SARS-CoV-1), and Middle East respiratory syndrome coronavirus (MERS-CoV). CoV belongs to the betacoronavirus class [37,43]. Phylogenetic analysis shows that SARS-CoV-2 belongs to the same subgenus as CoVs that caused the outbreak of severe acute respiratory syndrome (SARS) in 2002–2004 [44] addition, the SARS-CoV-2 sequence is similar to CoVs isolated from bats [45]. The SARS-CoV-2 genome has an 89% similarity in homology compared to the ZXC21 bat coronavirus and an 82% similarity to SARS-CoV-1 [6,46]. Thus, a hypothesis was deduced that SARS-CoV-2 originated from bats, which mutated and became infectious to humans [39,47].

**Figure 2:**
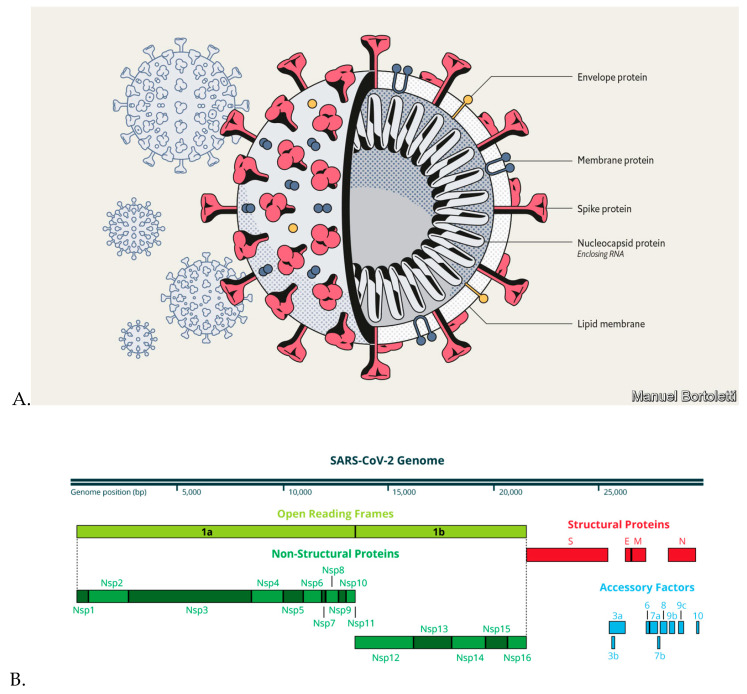
(**A**) The structure of severe acute respiratory syndrome coronavirus 2 (SARS-CoV-2) (https://www.economist.com/briefing/2020/03/12/understanding-sars-cov-2-and-the-drugs-that-might-lessen-its-power) and (**B**) its genome [51].

The genome of SARS-CoV-2 contains 14 open reading frames (ORFs) encoding 27 proteins (Figure 2). The 5ʹ terminus encodes for 15 nonstructural proteins collectively involved in virus replication and possibly in immune evasion, while the 3ʹ terminus encodes for structural and accessory proteins [42,48]. The presence of a spike protein (S protein), which resembles a nail or an arrow on the surface of this virus, makes the structure even more unique than others. This S protein attaches to the angiotensin-converting enzyme (ACE) 2 receptors on the surface of host respiratory cells [49,50].

## 4. Angiotensin-Converting Enzyme 2 (ACE2) 

SARS-CoV-2 uses the angiotensin-converting enzyme (ACE) 2 receptor for entry into target cells. ACE2 is largely expressed by epithelial cells of the lung, kidney, heart, blood vessels, and intestine. ACE and ACE2 belong to the ACE family of dipeptidyl carboxydipeptidases, and they have distinct functions. ACE converts angiotensin I into angiotensin II, which in turn binds and activates angiotensin II receptor type 1 (AT1R). This activation leads to vasoconstrictive, pro-inflammatory, and pro-oxidative effects [52]. ACE2 exists in two forms: a soluble form that represents the circulating ACE2, and a structural transmembrane protein with extracellular domain that serves as a receptor for the spike protein of SARS-CoV-2. The latter is a polypeptide composed of 805 amino acids [53]. This molecule is an inseparable part of a type 1 membrane protein that breaks down the main residue (a single hydrophobic molecule) on the carboxy C-terminal of any bound substrate [54]. ACE2 hydrolyzes the C-terminal domain of leucine from Ang I to produce non-peptides angiotensins 1–9 that can be converted into heptapeptides by ACE and other peptidases. Furthermore, ACE2 can directly reduce angiotensin II to angiotensins 1–7 [55]. Angiotensins 1–7 work on the Mas receptors to relax blood vessels and exhibit anti-proliferation and anti-oxidative activities. ACE2/angiotensins 1–7/Mas formed by the participation of angiotensins 1–7 can attack certain parts of ACE–angiotensin II–AT1R, with functions in maintaining the balance of the body [55,56].

The binding of SARS-CoV to the ACE2 receptor regulates the cellular expression of the receptor, and the binding process induces internalization, which depends on clathrin [57]. ACE2 not only facilitates the invasion and rapid replication of SARS-CoV, but it is also used by the cell membrane, thus damaging angiontensin II, which results in acute damage of lung tissues [58]. Because the lungs are the main target organs for COVID-19 infection, early onset of respiratory symptoms is common among patients [59]. The results of the study conducted by Imai et al. [60] showed that blocking the renin–angiotensin signaling pathway could relieve severe acute lung injury caused by SARS-CoV-2.

**Figure 3:**
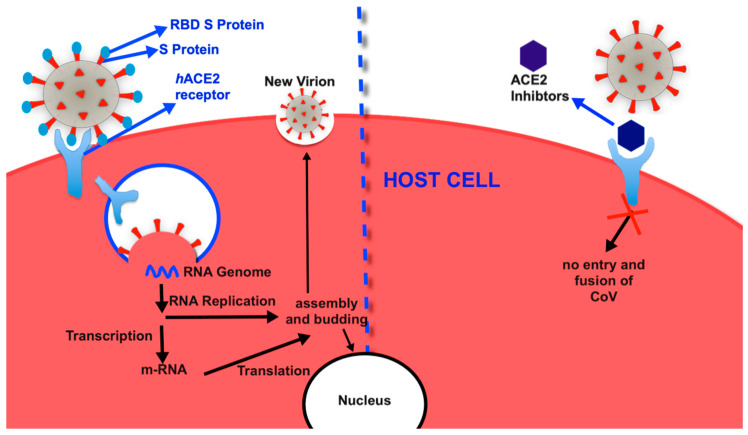
The life cycle of SARS-CoV. The spike (S) protein of SARS-CoV binds with the angiotensin-converting enzyme 2 (ACE2) receptor to enter host cells and release the RNA genome into the target cells. Structural and nonstructural proteins of CoV and the RNA genome assemble into virions, which are released from target cells.

**Figure 4:**
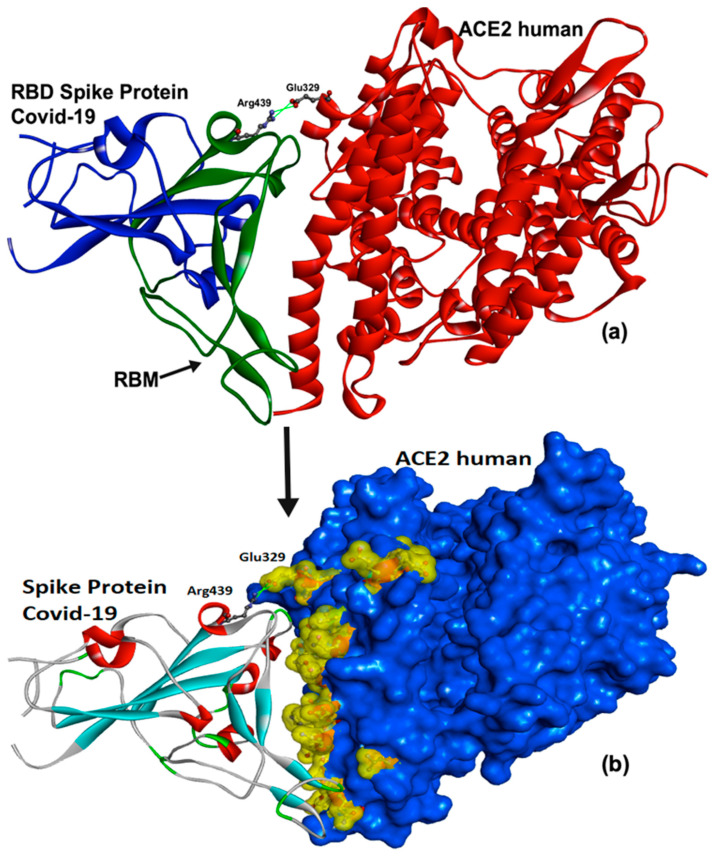
(**a**) Structure of the receptor-binding domain (RBD) of the S protein in SARS-CoV-2 (blue and green ribbons) complexed with human ACE2. The green ribbon denotes the receptor-binding motif (RBM) within amino-acid residues 424–494 or 438–504 [62,64]. (**b**) The active site of ACE2 (yellow color) that directly interacts with the RBD of the S protein of SARS-CoV-2. The interaction between the S protein of SARS-CoV-2 and hACE2 is stabilized by a hydrogen bond (green lines) between Arg439 (S protein SARS-CoV-2) and Glu329 (hACE2). The figure was created using Discovery Studio Biovia through visualization of the Protein Data Bank (PDB) structure 6VW1 [65].

SARS-CoV-2 attaches to human ACE2 through the binding of spike (S) proteins, as shown in Figure 3 [61]. The S protein of SARS-CoV-2 contains S1 and S2 subunits. The S1 subunit (Figure 4) consists of a receptor-binding domain (RBD) that is responsible for binding with the host ACE2, and the S2 subunit facilitates membrane fusion in the host cells [62,63]. The RBD contains a loop-binding pocket (residue 424–494 or 438–506), which is called the receptor-binding motif (RBM) [62,64]. The RBM cleaves the ACE2 receptor so that SARS-CoV can enter the host cells. After SARS-CoV binds to ACE2, the S2 subunit facilitates membrane fusion in the endosomal plasma through conformational change, thereby releasing the RNA genome into the target cells. After transcription and translation, the structural and nonstructural proteins of CoV and the RNA genome are further assembled into virions, which are transported through vesicles and released from target cells.

### The Active Site of hACE2 as the Therapeutic Target of COVID-19

The amino-acid sequence of SARS-CoV-2 has a 76.5% similarity to that of SARS-CoV, and their S proteins are quite homologous [66,67]. As shown in Figure 4, the RBD of the S protein of SARS-CoV-2 is located within amino-acid residues 318–510 (left side), containing the RBM (green ribbon), which is on the surface, right in front of ACE2. Arg439 of the RBM in SARS-CoV-2 and Glu329 of ACE2 interact and form a bridge to stabilize the complex. Based on the interaction of ACE2 with the S protein in SARS-CoV-2, antibodies or small molecules can be used to target and inhibit SARS-CoV-2 replication through inhibition of the ACE2 receptor. The S protein, thus, loses its partners to enter the host cell, as illustrated on the right side of Figure 4. ACE2 can be a target for inhibiting the entry of SARS-CoV-2 into the host cell because the binding affinity of the S protein of SARS-CoV-2 to the ACE2 receptor is 10–20-fold stronger than that of the S protein of SARS-CoV [68,69,70].

Han et al. identified the residues of ACE2 that directly interact with the RBD of the SARS-CoV-2 S protein. The residues involved are Gln24, Thr27, Lys31, His34, Glu37, Asp38, Tyr41, Gln42, Leu45, Leu79, Met82, Tyr83, Asp90, Gln325, Glu329, Asn330, Lys353, and Gly54. They also determined that Glu22, Glu23, Lys26, Asp30, Glu35, Glu56, and Glu57 are important in the interaction. Notably, Lys26 and Asp30 play a critical role in the interaction of the RBD S protein of SARS-CoV; thus, Han et al. concluded that these residues have the potential to be developed as a target for entry inhibitors [71]. Moreover, Gln325/Glu329 and Asp38/Gln42 of ACE2 are key binding sites that form hydrogen bonds with Arg426 and Tyr436 of the S protein SARS-CoV-2 [72]. These critical residues are also present in the S protein of SARS-CoV-2 with a similar sequence [73]. Therefore, the residues can be used as primary target active sites of ACE2 inhibitors. We hypothesize that, if the inhibitors selectively bind to this active site (shown in yellow color in Figure 2), then they might be able to inhibit the S protein of SARS-CoV-2 from interacting with hACE2. Guy et al. [74] hypothesized that the residues of the ACE2 binding pocket differ slightly from those of the active site of ACE2 (isolated from pig kidney tissue). However, the types of amino acids involved are nearly the same.

## 5. Inhibitors of ACE2

### 5.1. Synthetic Compounds of ACE2 Inhibitors

Research on ACE2 inhibitors or blockers is still lacking, and only very few drugs are currently available in the clinics. However, ACE1 inhibitors, such as losartan, are widely marketed. Several countries use ACE1/ARB, such as losartan and telmisartan, to reduce the aggressiveness and mortality of COVID-19. Kuster et al. proposed that ACE1 therapy should be continued or initiated on patients with a history of heart failure, hypertension, or myocardial infarction [75] Zhang et al. [76] found that, among patients with hypertension who were hospitalized with COVID-19, inpatient treatment with ACEI/ARB was associated with a lower risk of death from all causes compared to non ACEI/ARB users. ARB is widely used to treat hypertension, and the use of this drug clinically provides exceptional tolerance for several groups treated with this class of drugs. In addition, the profile of side effects is described as “like a placebo”. ARBs are most suitable for antagonizing the proinflammatory effects of angiotensin II in patients with a recent positive COVID-19 test; thus, this compound may have the best pharmacological properties for this indication. From the comparative analysis of available ARBs, telmisartan has traits that make it the best compound [77].

Angiotensin receptor blockers (ARBs) have effects similar to angiotensin-converting enzyme (ACE) inhibitors, but ACE inhibitors act by preventing the formation of angiotensin II rather than blocking the binding of angiotensin II to muscles in blood vessels. ARB is used to control high blood pressure, treat heart failure, and prevent kidney failure in diabetics. Therefore, angiotensin receptor blockers (ARBs; such as losartan, valsartan, telmisartan, etc.) can be a new therapeutic approach to block the binding and, hence, the attachment of SARS-CoV-2 RBD to cells that express ACE2, thereby inhibiting their infection of the host cell [78].

In the past 20 years, MLN-4760 (imidazole) [79,80,81], captopril derivative [82,83], DX600 and TAPI-2 peptide [84,85], losartan and its derivatives (benzimidazole [56,82,86,87], chloroquine and its derivatives (quinolone) [88], diminazene aceturate [89], cepharanthine (alkaloid) [75], thiorphan (palmitoyl) [87], and *N*-(2-aminoethyl)-1 aziridineethanamine (amino ethyl) [90] were discovered to have potential as ACE2 inhibitors. However, caution should be taken because, although ACE1 inhibitors (such as captopril, enalapril, and lisinopril) and angiotensin II receptor blockers (ARB) (such as olmesartan, losartan, candesartan, and valsartan) do have inhibitory effects on ACE2 [91], several studies showed that these drugs can increase the ACE2 blood level [86], which will likely increase the risk of contracting SARS [92]. This drawback means that the search for new and effective drugs is even more pressing in order to combat the infection of this deadly virus, and we believe that natural products should be further explored in the quest to find suitable and effective drug candidates [92].

### 5.2. Natural Compounds Inhibiting ACE1 and ACE2 Receptors

The discovery of novel drugs from natural products helps to improve our understanding of diseases [93,94]. The active lead compounds from natural products can be further modified to enhance their biological activity in order to be developed as drug candidates [95,96]. Recent progress on natural products resulted in compounds being developed to treat viral infections [97]. Utomo et al. [98]. reported the biological activity of natural products in inhibiting SARS-CoV-2 using in silico methods. Islam et al. comprehensively reviewed studies on natural products with inhibitory activity against CoV.

Natural products such as flavonoids, xanthones, proanthocyanidins, secoiridoids, and peptides were reported to contain anti-ACE activity; however, further research is needed to confirm the findings [24]. Table 1 summarizes the natural compounds that were reported to have inhibitory effects on ACE1 and ACE2 receptors. From this table, we can conclude that flavonoids are the most researched with regard to ACE inhibition activity.

## 6. Flavonoids as ACE2 Inhibitors

**Figure 5:**
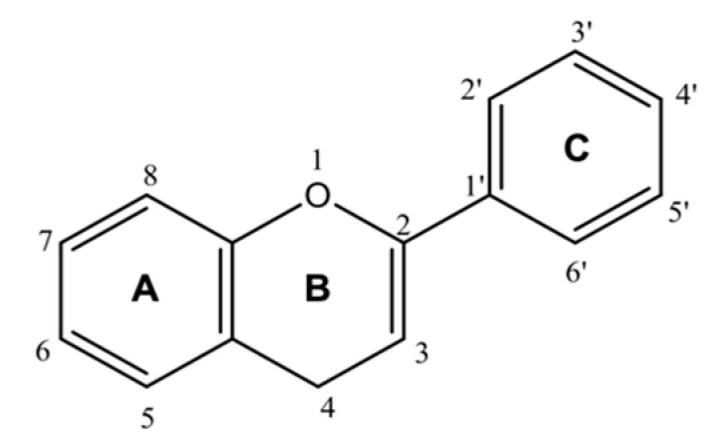
Flavan core of flavonoids.

**Figure 6:**
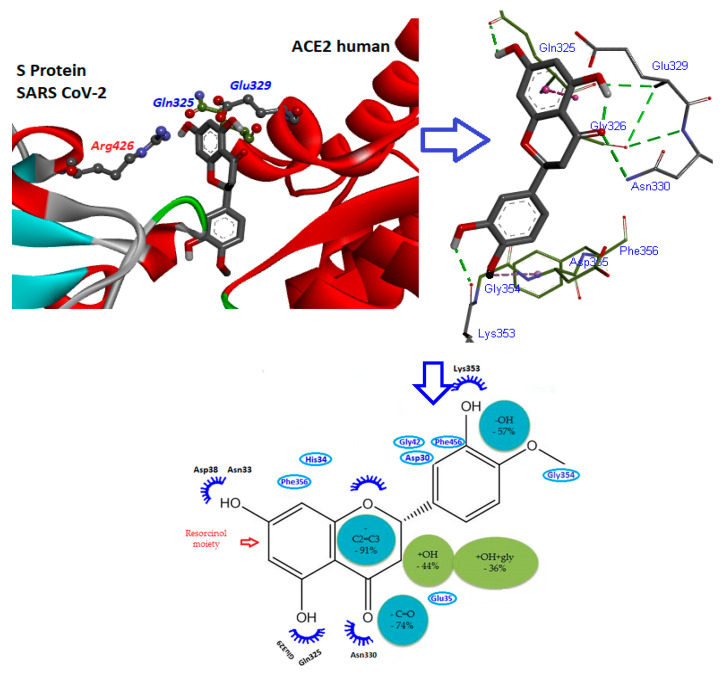
Overview of the most important functional groups of flavonoids that might be involved in ACE2 inhibition.

Flavonoids are an important class of natural products with several subgroups, including chalcones, flavones, flavonols, and isoflavones [109]. Flavonoids contain a flavan core with a 15-carbon skeleton. There are two benzene rings (A and C rings), connected by a heterocyclic pyran ring (B ring). The three cycles or heterocycles in the flavonoid backbone are generally called rings A, B, and C, as shown in Figure 5. The B ring comprises a C2–C3 double bond and carbonyl groups that play an important role in the biological activities. The hydroxyl groups (3′ and 5′ positions) of the C ring, as well as the hydroxyl groups of the A ring (7 and 5 positions), are known to be responsible for the radical scavenging activity of flavonoids [103]. The most important functional groups of flavonoids that might be involved in ACE2 inhibition are illustrated in Figure 6.

As can be seen in Figure 6, the resorcinol molecule has two hydroxyl groups in its aromatic ring structure, and they are located at *meta*-positions with respect to each hydroxyl group. The high reactivity of the resorcinol structure is primarily associated with the location of these two hydroxyl groups in the benzene ring [110]. The resorcinol moiety of ring A might play a role in ACE2 inhibition, as this group might disrupt hydrogen bonds between Glu329/Gln325 of ACE2 and Arg426 of the S protein of SARS CoV-2, which form a salt bridge to stabilize their interaction [72,73].This hydrophobic interaction occurs in ring C with some non-polar amino acid residues such as Gly354, Asp355, and Phe356 [111].

As summarized in Table 1, flavonoids have potential as ACE1 and ACE2 inhibitors. Studies on flavonoids for anti-SARS-CoV activity were widely published. For example, myricetin inhibits viral replication by affecting the ATPase activity of SARS-CoV [112]. Other flavonoids reported to have anti-SARS-CoV activity include kaempferol [113], luteolin [114], quercetin, daidzein, EGCG, GCG, and herbacetin [115,116]. Quercetin functions as an inhibitor or noncompetitive inhibitor of 3-chymotripsin-like protease (3CLpro) and papain-like protease (PLpro) [117]. Luteolin inhibits furin proteins which are known to be some of the enzymes that break down the S protein of SARS-CoV, as reported in the Middle East respiratory syndrome (MERS) [114]. Kaempferol functions as a noncompetitive inhibitor of 3CLpro and PLpro [117]. Hesperidin inhibits the interaction between the RBD of the S protein of SARS-CoV-2 and the ACE2 receptor in humans; thus, it was also predicted to potentially inhibit the entry of SARS-CoV-2 [118].

## 7. Mode of Action of Flavonoids

Polyphenolic compounds, including flavonoids, terpenoids, hydrolysable tannins, xanthones, procyanidin, and caffeoylquinic acid derivatives, were discovered to be effective natural ACE inhibitors [119,120]. Table 2 summarizes the studies on plant extracts rich in flavonoids used as ACE2 inhibitors.

A number of epidemiological studies suggested a negative relationship between the consumption of flavonoid drugs and the development of various diseases. Flavonoids with typical structures can interact with enzyme systems involved in important pathways, showing effective poly-pharmacological behavior. Thus, it is not surprising that the relationship between chemical structures and their activities was widely studied [124]. The presence of C2=C3 double bonds in conjugation with C4 carbonyl groups of certain groups on flavonoids, as well as hydroxylation patterns, especially the catechol portions of ring B, methoxyl groups, and fewer saccharide bonds, provides higher antioxidant properties. The mechanism might involve planarity, which contributes to the shifting of electrons across the next molecule and affects the dissociation constant of the hydroxyl phenolic group, such that the whole molecule can bind to the target molecule, similar to an enzyme that matches the pattern [125].

Guerrero et al. [103] comprehensively analyzed different flavonoids to determine the functional groups responsible for inhibiting ACE. Quantitative structure–activity relationship (QSAR) modeling was conducted, and the lack of the B ring in the flavonoid skeleton was shown to reduce the inhibitory activity of ACE by up to 91%. The absence of carbonyl groups in the B ring also reduced the inhibitory activity of ACE by 74%. The 3-OH, 3′-OH, and 5′-OH groups are important since the loss of these groups reduced inhibitory activity by 44%, 57%, and 78% [103], respectively, as shown in Figure 6. These groups also play an important role in inhibiting neuraminidase receptors of the influenza A viruses (H1N1 and H3N2) [126]. Other studies also reported that losing the 3-OH group significantly reduced flavonoid antioxidant [127] and anti-CoV activities [115]. We also observed that 3-OH and catechol of the C ring moiety of catechin formed strong hydrogen bonds with H1N1 neuraminidase [126]. Hošek and Šmejkal [128] reported that these functional groups play an important role in anti-inflammatory activity against the receptor target of inflammation. Moreover, hesperidin was also reported as an ACE2 inhibitor since it can interact with the RBD of the S protein SARS-CoV2 and hACE2 interface. The dihydroflavone moiety of hesperidin was predicted to be parallel to the β-6 RBD S protein sheet, while the sugar moiety fits into a shallow hole in the direction away from ACE2 [118].

The most critical mechanism of flavonoids as antioxidant, anti-inflammation, anticarcinogenic, and antiviral compounds is the protection of the body against reactive oxygen species (ROS) [129,130]. ROS interferes with cellular function through the role of lipid peroxidation, resulting in damaged cell membranes. An increase in ROS production during tissue injury is due to the depletion of endogenous scavenger compounds [131,132]. Flavonoids have a role as endogenous scavenging compounds [133]; thus, flavonoids can prevent inflammation or repair cell damage by scavenging ROS. The interaction between flavonoids and hydrophilic amino-acid residues of protein targets with strong affinity is suggested to be a mechanism of flavonoids in repairing cell damage [130,134].

Based on these findings, we believe that there is a strong relationship among the ACE2 inhibition, anti-inflammation, and antioxidant activities of flavonoids. However, the correlation among these three activities needs to be clarified through comprehensive in vitro and in vivo evaluation.

## 8. Perspectives and Overall Conclusion

The renin–angiotensin system (RAS) controls the homeostatic function of the vascular system. The two important enzymes involved in the RAS system, ACE1 and ACE2, function in accommodating rapid but coordinated feedback to any specific situation in the body that may disturb the system balance [135]. Their function is indispensable; hence, the choice to modulate these receptors for other health conditions, such as against the current COVID-19 infection, would have to be done in a careful manner.

Based on the information put forth in this review, it can be concluded that ACE2 could be a key receptor to combat COVID-19 infection. The inhibition of hACE2 may prevent the S protein of SARS-CoV-2 from fusing and entering host cells. However, as both RAS enzymes influence each other, inhibition of ACE2 alone in this case would lead to an increase in Ang II blood levels and a parallel reduction in the blood concentration of vasodilators angiotensins 1–7. In such a case, any disturbance in circulation homeostasis would not be corrected rapidly due to the absence of angiotensins 1–7. This would be a health risk, especially to susceptible patients such as the elderly and patients with underlying CVS-related medical conditions. Ironically, these are the group of people that would have a higher risk of contracting severe COVID-19 infection.

The discovery of ACE2 as a part of the RAS is relatively new; however, some evidence shows that ACE2 could be more important than ACE1 in the modulation of the whole system. Although the morphology of ACE1 and ACE2 receptors shares huge similarities, ACE inhibitors (ACEis) cannot inhibit ACE2 receptors. Hence, the currently available ACEis are not as useful as ACE2 inhibitors [135]. This means that the structure of ACEis cannot be used as a building block in the design of ACE2 inhibitors. A new and fresh approach should be taken, and a comprehensive study of the receptor itself is needed.

Thus, this paper proposes to shift the focus in the design of ACE2 inhibitors toward flavonoids, which are an abundant group of compounds that can be found in many plants. The functional groups of flavonoids, such as the pyran moiety in the B ring and hydroxyl groups of the A ring (7- and 8-positions) and C ring (3-, 3′-, 4′-, and 5′-positions), may play an important role in their ACE2 inhibition. Preliminary research showed that Glu22, Glu23, Lys26, Asp30, Glu35, Glu56, and Glu57 of the hACE2 could be used as primary target sites in the design of an hACE2 inhibitor.

Flavonoids are synthesized by plants in response to microbial attacks; hence, their antibacterial and antiviral activities are expected. The wide variety of activities reported in the literature depends on the structures and side chains available in each flavonoid [127]. Despite the available data on the activity of certain flavonoids against ACE1 and ACE2 enzymes, as presented in Table 1, the studies were stopped at in silico or in vitro stages, and no further detailed studies are available. This could be due to some limitations surrounding the research on natural products, such as difficulties in obtaining a sufficient amount of substance through plant extractions or difficulties in the chemical synthesis of the flavonoids. However, the application of flavonoid-based scaffolds in the design of new ACE2 inhibitors could be a good approach. Based on the history of drug development, a combination between natural-based products and chemical synthesis is able to produce potent and effective medications, such as the anticancer drugs vincristine and vinblastine. This could be an approach to bring forward natural-based products for human use.

## Figures and Tables

**Table 1:** Bioactive compounds reported to inhibit ACE1 and ACE2 in the literature.

No	Inhibitors	Derivates	Plants	Methods	Years	Source
1.	Luteolin	Flavonoid	*Ailanthus* *excelsa*	In vitro using **ACE2** via Elbl and Wagner methods	2007	[99]
2.	Kaempferol					
3.	Apigenin					
4.	Quercetin					
5.	Luteolin					
6.	Emodin	Anthraquinone	*Rheum officinale* *Polygonum multiflorum*	In vitro using **ACE2**	2007	[100]
7.	Chrysin	Flavonoid				
8.	Rhein	Flavonoid				
9.	Delphinidin	Flavonoid	*Hibiscus sabdariffa*	In vitro **ACE** Inhibition assay	2010	[101]
10.	Cyanidin	Flavonoid				
11.	Apigenin	Flavonoid	*Apium graveolens*	In vitro using **ACE2** isolated from kidney	2010	[102]
12.	Rhoifolin	Flavonoid	*Rhus succedanea*	**ACE** activity was measured by a fluorometric assay	2012	[103]
13.	Rutin and Quercetine		*Fagopyrum tataricum*			
14.	Nicotianamine	Peptide	*Glycine max*	In vitro using internally quenched fluorogenic (IQF) substrate for **ACE2**	2015	[104]
15.	Quercetin	Flavonoid	*Actinidia macrosperma*	In vitro using a fluorescence-based biochemical assay against **ACE** enzyme	2018	[103,105]
16.	Catechin					
17.	Quercetin					
18.	Epigallocatechin					
19.	Epigallocatechin gallate					
20.	Ferulic acid	Phenolic acid				
21.	Chlorogenic acid					
22.	Isoferulic acid					
23.	Caffeic acid					
24.	δ-Viniferin	Flavonoid	*Vitis vinifera*	Virtual screening against **ACE2** using Autodock Vina	2020	[106]
25.	Myritilin	Flavonoid				
26.	Myricitrin	Flavonoid				
27.	Taiwanhomoflavone A	Flavonoid	*Cephalotaxus wilsoniana*			
28.	Lactucopicrin 15-oxalate	Sesquiterpene lactone	*Lactuca virosa*			
29.	Nympholide A	Flavonoid	*Nymphaea lotus*			
30.	Afzelin	Flavonoid	*Cornus macrophylla*			
31.	Biorobin	Flavonoid	*Acalypha indica*			
32.	Phyllaemblicin B	sesquiterpenoid	*Phyllanthus emblica*			
33.	Baicalin	Flavonoid	*Scutellaria* *baicalensis*	Using spectroscopy method to determine renin and **ACE** activities		[107]
34.	Hesperetin	Flavonoid	*Citrus* *aurantium*	Virtual Screening against **ACE2** using molecular docking	2020	[108]
35.	Baicalin	Flavonoid	*Scutellaria* *baicalensis*			
36.	Scutellarin	Flavonoid				
37.	Glycyrrhizin	Sesquiterpene	*Glycyrrhiza radix*			
38.	Curcumin	Curcuminoids	*Curcuma xanthoriza*	Virtual Screening against **ACE2** using MOE molecular docking	2020	[98]
39.	Tangeretin	Flavonoid	*Citrus aurantifolia*			
40.	Nobiletin					
41.	Naringenin					
42.	Brazilein	Flavonoid	*Caesalpinia sappan*			
43.	Brazilin					
44.	Galangin	Flavonoid	*Alpinia galanga*			
45.	Acetoxychavicol acetate (ACA)	ACA derivatives				

**Table 2:** Plants with potential ACE2 receptor inhibition activity.

Name	Inhibition Approach	Effective Compound	Inhibition Potential (IC_50_/EC_50_) *	ADME	Reference
*Rheum* *officinale* (rhubarb)	Viral spike protein and human ACE2 receptor inhibitor	Emodin	1–10 µM/mL	HIA: 85.74 Caco2: 20.30 PPB: 88.75 BBB: 0.37	[119]
*Reynoutria* *multiflora* tuber	Viral spike protein and human ACE2 receptor inhibitor	Emodin	1–10 µM/mL	HIA: 85.74 Caco2: 20.30 PPB: 88.75 BBB: 0.37	[119]
Citrus accumulate	Viral spike protein and human ACE2 receptor inhibitor	Naringenin	Not yet reported	HIA: 87.31 Caco2: 10.52 PPB: 100 BBB: 0.59	[100]
*Citrus aurantium* and Citri Reticulatae Pericarpium	Viral spike protein and human ACE2 receptor inhibitor	Hesperetin	Not yet reported	HIA: 87.19 Caco2: 7.003 PPB: 96.79 BBB: 0.22	[121]
*Scutellaria baicalensis* Georgi	Viral spike protein and human ACE2 receptor inhibitor	Baicalin	2.24 mM	HIA: 32.42 Caco2: 11.55 PPB: 75.69 BBB: 0.02	[108]
Citrus	Viral spike protein and human ACE2 receptor inhibitor	Neohesperidin	Not yet reported	HIA: 8.80 Caco2: 7.07 PPB: 44.05 BBB: 0.02	[100]
Citrus	Viral spike protein and human ACE2 receptor inhibitor	Nobiletin	Not yet reported	HIA: 98.89 Caco2: 54.05 PPB: 85.16 BBB: 0.044	[100]
*Erigeron breviscapus (Vant.)*	Viral spike protein and human ACE2 receptor inhibitor	Scutellarin	Not yet reported	HIA: 13.45 Caco2: 10.13 PPB: 72.90 BBB: 0.029	[121]
Soya bean (*Glycine max*)	Viral spike protein and human ACE2 receptor inhibitor	Nicotinamine	84 nM	HIA: 92.94 Caco2: 20.36 PPB: 2.02 BBB: 0.33	[122]
Licorice root (*Glycyrrhiza radix*)	Viral spike protein and human ACE2 receptor inhibitor	Glycyrrhizin (saponin)	Not yet reported	HIA: 38.22 Caco2: 20.37 PPB: 88.72 BBB: 0.055	[121]

* Inhibitory concentration (IC_50_) is an indication of the concentration (μM or ug/mL) where the activity of the viral protein is reduced by up to 50%. Effective concentration (EC_50_) is the indication of the concentration (μM or μg/mL) where the activity of the viral growth is reduced by up to 50%. Absorption, distribution, metabolism, and excretion (ADME): human intestinal absorption (HIA) values of 20–70% indicate sufficiently absorbed compounds, and 70–100% HIA values indicate well-absorbed compounds. Caco-2 values <4 indicate low drug permeability, values from 4–70 indicate moderate permeability, and values >70 indicate high permeability. Plasma protein binding (PPB) values >90% indicate strong chemical bonds, while values <90% indicate weak chemical bonds. Blood–brain barrier (BBB) values between 2.0 and 0.1 indicate a moderate absorption rate in the central nervous system (CNS), while BBB values <0.1 indicate a low absorption rate in the CNS [123].

## Abbreviations

SARS-CoV: severe acute respiratory syndrome coronavirus, ACE1: angiotensin-converting enzyme 1, ACE2: angiotensin-converting enzyme 2, ARBs: angiotensin receptor blockers, 3CLpro: 3-chymotripsin-like protease, PLpro: papain-like protease, Mpro: main protease, MERS: Middle East respiratory syndrome, AT1R: activates angiotensin II receptor, ORFs: open reading frames, FDA: Food and Drug Administration, CRS: cytokine release syndrome, RBD: receptor-binding domain, RNA: ribonucleic acid, IQF: internally quenched fluorogenic, MOE: molecular operating environment, EGCG: epigallocatechin gallate, GCG: gallocatechin gallate, IC_50_: the half maximal inhibitory concentration, EC_50_: the half maximal effective concentration, ADME: absorption, distribution, metabolism, and excretion, HIA: human intestinal absorption, PPB: plasma protein binding, BBB: blood–brain barrier, CNS: central nervous system, QSAR: quantitative structure–activity relationship, ROS: reactive oxygen species, RAS: renin–angiotensin system.
